# Selfie-related concepts and behaviors among Egyptian and Saudi nursing students: a comparative study

**DOI:** 10.1186/s43045-021-00133-5

**Published:** 2021-09-16

**Authors:** Marwa Abd El-fatah Ali El-slamoni, Hanem AbdElkhalek Ahmed, Azza Elsayed Abdelfatah Arafat

**Affiliations:** 1 Department of Psychiatric and Mental Health Nursing, Faculty of Nursing, Zagazig University, Zagazig, Egypt; 2 Department of Psychiatric and Mental Health Nursing, College of Applied & Medical Science, Taif University, Taif, Kingdom of Saudi Arabia

**Keywords:** Self-esteem, Body image, Selfies habits, Motives, Attitudes, Egyptian, Saudi, Nursing

## Abstract

**Background:**

Over the last 10 years, social media has become an integral facet of modern society. Self-presentation and body satisfaction are related to social media and its impact on users’ levels of well-being and self-esteem. This study aimed to compare selfie-related concepts (self-esteem and body image) and behaviors (selfie habits and patterns, the motives for using the selfie, and attitude about selfie) between samples of Egyptians and Saudis student nurses. To attain this research aim, a comparative research design study was conducted between 7th of October and 5th of November 2020. This study was conducted in the two countries: Kingdom of Saudi Arabia (KSA) (College of Nursing, Taif University) and Egypt (Faculty of Nursing, Zagazig University). A sample of 300 students was included in two countries: KSA and Egypt. Socio-demographic data sheet, patterns of selfie use scale, self-confidence questionnaire (SCQ), and body image scale.

**Results:**

Results revealed that the Egyptian students were higher in number of selfies per day than Saudi students and the majority of Saudi group like to put their selfies on Instagram.

**Conclusion:**

The current study deduced that Egyptian students as regard selfie habits and patterns had a higher number of selfies per day than Saudi students, also the majority of the Saudi group as regard selfie habits like to put their selfies on Instagram with a statistically significant difference. As regards the motives for using the selfie, the reasons for taking selfies were significantly higher in Saudi nursing students than in Egyptian students regarding depression and sadness. As well, significantly higher in Egyptian than in Saudi nurse students was related to motivates that their selfies on social media often contain comments or answers, the relationship between the number of selfie-taking and gender was a statistically significant difference between the two genders in both Egyptian and Saudi groups with increasing taking selfie among Egyptian females and Saudi males.

## Background

A selfie is a photograph taken by a person for himself without the help of others, using a fast self-portrait taken with a smartphone or webcam that can be instantly posted and spread on social media [1]. “Selfie,” according to Oxford Dictionaries [2], is the word of the year for 2013, and the term was created to describe the act of taking a self-portrait and posting it on social media. With the introduction of numerous smart devices and social media, taking and posting selfies has become common place [3]. Selfies have almost become a regular habit for many people all over the world, and social media networks have become the primary locations for those people to share their selfies [4].

Selfies have become increasingly common, particularly among teenagers and young adults. Selfie-taking, on the other hand, is more than just taking a picture; it may also include color and contrast adjustments, background changes, and other effects before uploading the image to a social media site. Selfies are a self-centered activity that enables users to define their individuality and significance, and they are linked to personality traits like narcissism [5].

Selfies are a double-edged sword. For some people, taking selfies boosts their self-esteem, while for others selfies are the source of their misery and insecurity about their appearance. Most adolescents devote a significant amount of time and money to appear desirable to others and, as a result, improve their self-esteem [6]. Selfies have become an addiction among college students and have become a popular theme in their lives [7]. Selfies can make certain people feel good about themselves if they share them on social media sites [8].

Self-esteem is a general positive or negative assessment of oneself. One option to address self-esteem demands is to utilize social media to communicate interpersonally. This can give an incentive for persons with low self-esteem to participate in public activities with less risk of shame and social anxiety [9]. High self-esteem is seen to be a significant predictor of relationship pleasure and satisfaction [10]. Because they highlight what young people are going through, selfies might boost self-esteem and self-efficacy. Selfies can help improve self-presentation by publishing socially valued content [11], whereas another study found that having a habit of uploading selfies can have a negative impact on one’s self-esteem [12].

Posting selfies allows people to express their own identity and social relationships; other psychological factors can lead to various types of selfie behaviors [13]. Attitudes toward selfie-taking have been analyzed in three countries by Katz and Crocker [14]. The importance of self-presentation and identification in selfie creation, as well as the necessity for peer evaluation, was established in their research. Selfies also allow people experiment with their looks, accessories, and surroundings.

Self-evaluations of many distinct bodily traits, such as skin tone, dimensions, and size, are all part of body image. Body image is important because it encompasses social and cultural comparisons that can affect a person’s impression of physical attractiveness and self-worth [15]. Objectification theory states that women are more likely to be considered as physical and sexual objects whose social significance may be inferred from their physical appearance, despite the fact that body image is significant for both men and women. As a result, women are more prone to engage in self-objectification behaviors that reinforce the link between their physical bodies and their feeling of self-worth [16].

Body image has a significant impact on the creation of self-concept, which impacts people’s perceptions of themselves and others during adolescence. Further research found that body image dissatisfaction is closely linked to depression, eating disorders, and low self-esteem [17, 18]. Certain study revealed that people who were more satisfied with their body image posted more selfies to Instagram confidently showing off [19].

## Significance of the study

Researchers are interested in studying selfies since the number of selfies taken has increased by 17.00% since 2012 [20]. Body image is a complex combination of inner biological and psychological factors that influence persons’ self-esteem and mental health over the course of their lives. As the phenomenon of taking and posting selfies has grown in popularity among people all over the world, a number of studies have looked into it from various socio-psychological perspectives. So the researchers focused on self-esteem and body image among Egyptian and Saudi nursing students in the current study and its relations with number of selfies and posting on social media.

## Aim of the study

This study aims to compare selfie-related concepts (self-esteem and body image) and behaviors (selfie habits and patterns, the motives for using the selfie, and attitude about selfie) between samples of Egyptians and Saudis student nurses

## Methods

### Research questions

- What are selfie behaviors (habits and patterns, the motives for using the selfie, and attitude about selfie) among Egyptian and Saudi nursing students?
- What are the levels of self-esteem and body image perception among Egyptian and Saudi nursing students?
- Is there a relationship between self-esteem and both number of selfies and posting selfies on social media among Egyptian and Saudi nursing students?
- Is there a relationship between body image and both number of selfies and posting selfies on social media among Egyptian and Saudi nursing students?

### Research design

A comparative research design was utilized in this study.

#### Subjects

A purposeful sample of 300 nursing students (150 Saudis and 150 Egyptians) according to the following inclusion criteria: both genders, between the ages of 18-24 years, from 1st grade to 4th grade, use selfies and posting selfies on social media, and agreed to participate in the current study.

#### Sample size

Assuming, percent of selfie 6-10 photos per day among Egyptian students was 10.7% and 23.5% Saudi students (pilot study). Confidence level is 95% with power of study 80%. Sample size was calculated using epi info version 6.04, is 150 in each group.

### Tools of data collection

Four tools was used for data collection

#### (Tool I): Socio demographic data and personal characteristics

A structured questionnaire was established by the researchers including questions about student’s age, gender, and grade.

#### (Tool II): Patterns of selfie use

The scale was developed by Matager and Kunduz [21] to assess selfie habits and patterns, motives for using the selfie, and attitudes about the selfie. It consisted 28 items classified into three subscales as follows: habits and patterns of selfie use (14 questions); the second, the motives for using the selfie (5 questions); and the third, the look and trends of the sample response about the selfie (9 questions).

#### (Tool III): Self-confidence questionnaire (SCQ)

It was developed by the researchers based on previous studies to measure general self-esteem which is the attitude you have toward yourself [1, 10, 12, 18]. It consisted of 15 items to cover a group of nursing students such as coordinate and organize my work, I have the ability to resist the problems that I encounter, I hold positive beliefs about myself, I have the ability to adapt to the social environment in which I live, I try to benefit from the experiences of the former, and I am not afraid of confronting social situations of any kind; these items are scored using Likert scale with 3 response choices for each; 0 = rarely, 1 = sometimes, and 2 = always.

#### (Tool IV): Body image scale

The body image measurement was developed by Saber [22] with (27) statements and covers two dimensions: The individual’s perception of his or her body, which is positive or negative, and includes the following statements: items: 1, 2, 4, 5, 6, 7, 9, 10, 12, 13, 16, 17, 19, 20, 21, 23, 24, 25, 26, and the individual’s awareness of his or her body through others such as family, friends, and colleagues, and includes the following statements: items: 3, 8, 11, 14, 15, 18, 22, 27. Each positive statement has three response alternatives: (3) “yes,” (2) “sometimes,” and (1) “not” (items: 1, 2, 7, 10, 11, 16, 22, 24, 25, and 27). In negative statements (items: 3, 4, 5, 6, 8, 9, 12, 13, 14, 15, 17, 18, 19, 20, 21, 23, 26), the scoring is reversed: (1) “yes,” (2) “sometimes,” and (3) “not.” The value of that medium was calculated to measure the image of the body is 67, so that the higher scores of 67 indicate the positive body image and the real and clear perception of the body’s image satisfaction. The lower scores indicate a negative body image and the wrong person’s perception of his body image dissatisfaction. The total score was calculated by adding all the statements, which ranged from 27 to 81.

### Pilot study

The researchers conducted a pilot study on 10% of the studied students before beginning the actual study. It was done to assess the study questionnaire clearness, easiness, and feasibility, as well as to estimate the required time to complete the questionnaire. Based on the pilot study results, some changes were made on the questionnaire mostly rephrasing and using simpler semantic for the statements. Students who took part in the pilot study were not included in the main study.

### Content validity and reliability

Validity of tools II, III, and IV were tested for their content by a jury of five experts in the field of psychiatric mental health nursing to ascertain relevance and completeness of the tools and the needed modifications were done. Tools II, III, and IV reliability was assessed by Cronbach’s alpha coefficient test through their internal consistency. They presented good level of reliability as follow: Pattern of selfie score (*α* = 0.81), self-confidence score (*α* = 0.87), and body image score (*α* = 0.92).

### Fieldwork

After obtaining the required permission to conduct the study, the researchers interviewed the selected Egyptian nursing students in their classes in order to attain their verbal consent to participate in the study after being informed about its aim. The selected students were divided into four groups (35 to 40 students for each group). The researchers interviewed students explained each statement to them then asked them to complete the questionnaire. Each group required about 30-45 min to be completed. As a result of pandemic COVID-19, the necessity of taking safety precautions such as physical distancing, wearing a mask, keeping rooms well ventilated, avoiding crowds, cleaning hands, and application of blended learning (division students to many groups, one group attended to college, and the rest studied online in rotation), data collection was completed in about 1 month (1 day/week for each group).

For Saudi students, an online survey tool was available in College of Nursing, Taif University, KSA, and then provided students with the link to the survey. The online survey tool was used among KSA students as a result of depending on complete online education at the time of data collection as a result of pandemic COVID-19. The study was completed between 7th of October and 5th of November 2020 among Saudi and Egyptian undergraduate nursing students.

### Statistical analysis

All data were collected, tabulated, and statistically analyzed using the statistical package for social sciences (SPSS) version 20.0 for windows (SPSS Inc., Chicago, IL, USA). Quantitative data were expressed as the mean ± SD and range, and qualitative data were expressed as absolute frequencies (numbers) and relative frequencies (percentages). Percent of categorical variables were compared using Chi-square test when appropriate. All tests were two-sided. *p* value < 0.05 was considered statistically significant (S) and *p* value ≥ 0.05 was considered statistically insignificant (NS).

## Results

Table 1 shows that, about half of Egyptian and Saudi nurses students in the study sample their ages ranged from 20-21 years (52.7% & 50.7 % respectively), less than three quarters of Egyptian and almost two-thirds of Saudi nurse students in the study sample were female (72.0% and 65.3% respectively). Additionally, slightly more than one quarter of Egyptian nurse students (26.0%) were in grade four and slightly more than one-third of Saudi nurse students (34.7%) in the study sample were in grade three.

**Table 1:** Comparison between Egyptian and Saudi nursing students as regards socio demographic characteristics (each group *n* = 150)

Socio demographic characteristics	Studied groups				*χ*^2^	*p* value
	Egyptian group (*n* = 150)		Saudi group (*n* = 150)			
	No.	%	No.	%		
Age (in years)						
• ≤ 19	57	38.0	48	32.0	4.4	0.11
• 20-21	79	**52.7**	76	**50.7**		
• ≥ 22	14	9.3	26	17.3		
Sex						
• Female	108	**72.0**	98	**65.3**	1.55	0.21
• Male	42	28.0	52	34.7		
Grades						
• Grade one	37	24.7	26	17.3		
• Grade two	37	24.7	28	18.7	5.99	0.11
• Grade three	37	24.7	52	**34.7**		
• Grade four	39	**26.0**	44	29.3		

*χ*^2^ Chi-square test of significant, *p* > 0.05 insignificant

Regarding selfie habits and patterns, Table 2 shows that the Egyptian nursing students usually use selfies (58.7%), like to take their selfies during their first outing and vacation (53.3%), prefer to take selfies in important places and locations (84.0%), take selfies in green spaces (75.3%), and prefer to take selfies with friends and in the garden (69.3%) statistically significantly higher (*p* < 0.05) than Saudi nursing students (32.0%, 31.3%, 64.0% and 37.3%, 44.7%, and 48.0% respectively). whereas the same table represented that, the Saudi nursing students like to put their selfies on Instagram (82.0%) and like to photograph their selfies and display on social media in funny and cute situations (90.0%) were statistically significantly higher (*p* < 0.05) than in Egyptian nursing students (60.0% and 80.0% respectively).

**Table 2:** Comparison between Egyptian and Saudi nursing students as regard selfie habits and patterns (each group *n* = 150)

Selfie habits and patterns	Studied groups				*χ*^2^	*p* value
	Egyptian group		Saudi group			
	No.	%	No.	%		
Do you use selfie?						
• Rarely	28	18.7	26	17.3	27.88	0.0001
• Always	34	22.7	76	50.7		
• Usually	88	**58.7**	48	32.0		
Time of using selfie						
• For month	42	28.0	37	24.7	0.43	0.51
• More than years	108	72.0	113	75.3		
Number of selfie per day						
• 1-5	121	**80.7**	103	68.7	6.28	0.04
• 6-10	14	9.3	27	18.0		
• > 10	15	10.0	20	13.3		
Who do you prefer to take your selfies with?						
• Alone	34	22.7	67	44.7	19.36	0.0001
• With friends	104	**69.3**	67	44.7		
• With Family members	12	8.0	16	10.6		
When taking your selfie, how would you like your photo to be?						
• Normal and natural	70	46.7	62	41.3	1.05	0.59
• Image enhancements	53	35.3	61	40.7		
• Use selfie apps	27	18.0	27	18.0		
Which shooting angles you prefer when taking a “selfie”?						
• Head tilt	44	29.3	57	38.0		
• Photo from high angle	76	50.7	54	36.0	6.57	0.04
• Shooting from the front center	30	20.0	39	26.0		
What do you prefer to take your selfie with?						
• Expensive collectibles	5	3.3	31	20.7		
• Luxurious buildings	14	9.3	34	22.7	37.83	0.0001
• Garden	104	**69.3**	72	48.0		
• Archeological sites	27	18.0	13	8.7		
When would you like to take “selfies”?						
• Working hour	4	2.7	19	12.7		
• During my first outing, vacation	80	**53.3**	47	31.3	21.33	0.0001
• Learning time	14	9.3	13	8.7		
• Spare time	52	34.7	71	47.3		
Which places you prefer to take your selfies?						
• Green spaces	113	**75.3**	56	37.3		
• High places	5	3.3	32	21.3	54.82	0.0001
• University	20	13.3	21	14.0		
• The house	12	8.0	41	27.3		
Do you share your selfies on social media?						
• Rare	45	30.0	50	33.3		
• Usually	74	49.3	69	46.0	.44	0.803
• Always	31	20.7	31	20.7		
Which social site would you like to put your selfies on?						
• Instagram	90	60.0	123	**82.0**		
• Facebook	36	24.0	12	8.0		
• Snapchat	21	14.0	8	5.3	24.54	0.0001
• Twitter	3	2.0	7	4.7		
What situations would you like to photograph yourself and display on social media?						
• Funny and cute	120	80.0	135	**90.0**		
• Sharp	23	15.3	0	.0	26.79	0.0001
• Seriousness	7	4.7	15	10.0		
Whichever one you prefer to take a selfie with most of the time?						
• Famous people	19	12.7	22	14.7		
• Animals	5	3.3	32	21.3	23.98	0.0001
• Important places and locations	126	**84.0**	96	64.0		

*χ*^2^ test of significance, *p* < 0.05 (significant)

About the motives for using the selfie, Table 3 shows that the nurse students share photos on social media (48.7%); selfies on social media often contain comments (56.0%), subjective desire is the motivation for using selfie (80.0%), and their reason for taking selfies is state of joy (58.7%), statistically significantly higher (*p* < 0.05) in Egyptian than in Saudi nurse students (40.0%, 45.3%, 62.7%, and 48.0% respectively).

**Table 3:** Comparison between Egyptian and Saudi nursing students as regards the motives for using the selfie (each group *n* = 150)

Motives for using the selfie	Studied groups				*χ*^2^	*p* value
	Egyptian group		Saudi group			
	No.	%	No.	%		
Why do you share selfies on social media?						
• Create more friendships	39	26.0	19	12.7		
• Share photos	73	**48.7**	60	40.0	29.17	0.0001**
• Increased reactivity	14	9.3	35	23.3		
• Get to know foreigners	0	.0	12	8.0		
• Make an impression on your photos	24	16.0	24	16.0		
Do your selfies on social media often contain comments or answers?						
• No	29	19.3	11	7.3		
• Sometimes	37	24.7	71	47.3	20.49	0.0001**
• Yes	84	**56.0**	68	45.3		
What were your motivations for using selfie?						
• Fame love	8	5.3	14	9.3		
• Draw attention	6	4.0	17	11.3	12.03	0.007**
• Proof of existence	16	10.7	25	16.7		
• Subjective desire	120	**80.0**	94	62.7		
Reasons for taking selfies						
• State of joy	88	58.7	72	48.0		
• Depression and sadness	4	2.7	22	**14.7**	14.1	0.001**
• Normal case	58	38.7	56	37.3		
What were the media motives for taking your selfies?						
• Introduce my friends to places	44	29.3	29	19.3	5.53	0.14
• Introduce your culture	15	10.0	24	16.0		
• Notify a message	13	8.7	16	10.7		
• See your diary	78	52.0	81	54.0		

*χ*^2^ test of significance, *p* < 0.05 (significant)

Regarding the attitudes of studied nurse students about the selfie, as displayed in Table 4, they see some people photographing themselves in places for gaining fame (46.7%), see that the phenomenon has become an addiction for some people (93.3%), do not see selfies are really a reflection of the realities people experience (77.3%), think that they can stop selfie for 1 day (79.3%), see selfie style is so prevalent today as fashionable times (48.0%), and think that their selfie was frequently depicted in front of people as normal (77.3%), these were significantly higher (*p* < 0.05) in Egyptian than in Saudi nurse students (41.3%, 75.3%, 58.7%, 46.7%, 36.0%, and 58.0% respectively), whereas the nurses’ opinion about the person who is trying to prove himself through the selfie is someone who likes to impose himself (54.7%), and may be giving up selfies in the future and placing it with other technologies (62.0%) significantly higher (*p* < 0.05) in Saudi than Egyptian nurse students (44.0% and 56.7% respectively).

**Table 4:** Comparison between Egyptian and Saudi nursing students as regards their attitudes about selfie (each group *n* = 150)

Attitudes of studied nurse students about the selfie	Studied groups				*χ*^2^	*p* value
	Egyptian group (*n* = 150)		Saudi group (*n* = 150)			
	No.	%	No.	%		
Do you think your selfie is frequently depicted in front of people?						
• Harass them	28	18.7	37	24.7	17.89	0.0001
• Beloved to them	6	4.0	26	17.3		
• It is normal for you	116	**77.3**	87	58.0		
How do you see some people photographing themselves in places?						
• Adventure and risk	69	46.0	49	32.7		
• Gain fame	70	**46.7**	62	41.3	19.56	0.0001
• Breaking the numbers of extraordinary people	11	7.3	39	26.0		
In your opinion, the person who is trying to prove himself through the selfie is:						
• Extraordinary person	26	17.3	34	22.7		
• Spineless	58	38.7	34	22.7	9.06	0.01
• Someone who likes to impose himself	66	44.0	82	**54.7**		
Do you see that the phenomenon has become an addiction for some people?						
• Agree	140	**93.3**	113	75.3	18.39	0.0001
• Disagree	10	6.7	37	24.7		
Do you see selfies are really a reflection of the realities people experience?						
• No	116	**77.3**	88	58.7	12.01	0.001
• Yes	34	22.7	62	41.3		
Can you think, if for one day, to stop selfie?						
• Yes	119	**79.3**	70	46.7	34.33	0.0001
• No	31	20.7	80	**53.3**		
Are you thinking of giving up selfie in the future and replacing it with other technologies?						
• Sure	55	36.7	36	24.0		
• Never	10	6.7	21	14.0	8.23	0.02
• May be	85	56.7	93	**62.0**		
In your opinion, selfie style is so prevalent today?						
• New imaging technologies	33	22.0	41	27.3		
• Banality	24	16.0	0	.0	42.65	0.0001
• Fashionable times	72	**48.0**	54	36.0		
• It is okay	21	14.0	55	**36.7**		
What is your own evaluation of the selfie phenomenon?						
• Positive	102	**68.0**	88	58.7	2.81	0.09
• Negative	48	32.0	62	41.3		

*χ*^2^ test of significance, *p* < 0.05 (significant)

Regarding self-esteem level of studied nurse students, as displayed in Table 5, high self-esteem (52.0%) was significantly higher (*p* < 0.05) in Egyptian than in Saudi nurse students (38.7%) as well, the positive body image was higher in Egyptian (51.3%) than in Saudi nurses’ students (45.3%), but with a statistically insignificant difference between them.

**Table 5:** Comparison between Egyptian and Saudi nursing students as regards self-esteem level and body image perception of each group (*n* = 150)

Self-esteem level and body image perception	Studied groups				*χ*^2^	*p* value
	Egyptian group		Saudi group			
	No.	%	No.	%		
Self-esteem Levels						
• High self-esteem	78	**52.0**	58	38.7	6.4	0.042
• Need support	68	45.3	83	55.3		
• Low self-esteem	4	2.7	9	6.0		
Body Image perception				.		
• Positive body image	77	**51.3**	68	45.3	1.08	0.3
• Negative body image	73	48.7	82	54.7		

*χ*^2^ test of significance, *p* < 0.05 (significant)

Table 6 shows that, as regards the relationship between number of selfies per day and gender, there were statistically significant differences between the two genders, in both Egyptian and Saudi groups, with increasing number of selfies per day for female Egyptian students and male Saudi students (*p* < 0.05). There were statistically insignificant relationships between number of selfies taken per day and self-esteem levels and body image perception among Egyptian and Saudi students.

**Table 6:** Relationship between the number of selfies Egyptian and Saudi students per days and their gender, self-esteem, and body image perception

Variables	Number of selfie per day for Egyptian students							Number of selfie per day among Saudi students						
	1-5 (*n* = 121)		6-10 (*n* = 14)		> 10 (*n* = 15)		No.	1-5 (*n* = 103)		6-10 (*n* = 27)		> 10 (*n* = 20)		No.
	No.	%	No.	%	No.	%.		No.	%	No.	%	No.	%	
**Gender**														
Female	80	74.1	14	13.0	14	**13.0**	108	79	80.6	14	14.3	5	5.1	98
Male	41	97.6	0	.0	1	2.4	42	24	46.2	13	25.0	15	**28.8**	52
*χ*^2^ (*p* value)	10.9 (0.004)							22.41 (0.0001)						
**Self-esteem level**														
High self-esteem	65	83.3	6	7.7	7	9.0	78	41	70.7	13	22.4	4	6.9	58
Need support	52	76.5	8	11.8	8	11.8	68	54	65.1	13	15.7	16	19.3	83
Low self-esteem	4	100.0	0	.0	0	.0	4	8	88.9	1	11.1	0	.0	9
*χ*^2^ (*p* value)	2.13 (0.7)							7.1 (0.13)						
**Body image perception**														
Positive body image	59	76.6	7	9.1	11	14.3	77	51	75.0	8	11.8	9	13.2	68
Negative body image	62	84.9	7	9.6	4	5.5	73	52	63.4	19	23.2	11	13.4	82
*χ*^2^ (*p* value)	3.24 (0.2)							3.4 (0.18)						

*χ*^2^ test of significance, *p* < 0.05 (significant)

As regards the relationship between sharing selfies on social media and gender, Table 7 reveals that statistically significant difference was detected between the two genders in both Egyptian and Saudi groups with increasing sharing selfies on social media among males (*p* < 0.05). There were statistically insignificant relationships between Egyptian and Saudi students who share selfies on social media and self-esteem levels and body image perception.

**Table 7:** Relationship between share selfies on social media among Egyptian and Saudi nursing students and their gender, self-esteem, and body image perception

Variables	Egyptian students share selfies on social media							Saudi students share selfies on social media						
	Rare		Usually		Always		No.	Rare		Usually		Always		No.
	No.	%	No.	%	No.	%.		No.	%	No.	%	No.	%	
**Gender**														
Females	39	36.1	53	49.1	16	14.8	108	38	38.8	46	46.9	14	14.3	98
Males	6	14.3	21	50.0	15	**35.7**	42	12	23.1	23	44.2	17	**32.7**	52
*χ*^2^ (*p* value)	11.2 (0.0004)							8.1 (0.02)						
**Self-esteem level**														
High self-esteem	21	26.9	38	48.7	19	24.4	78	15	25.9	31	53.4	12	20.7	58
Need support	23	33.8	34	50.0	11	16.2	68	31	37.3	33	39.8	19	22.9	83
Low self-esteem	1	25.0	2	50.0	1	25.0	4	4	44.4	5	55.6	0	.0	9
*χ*^2^ (*p* value)	1.84 (0.8)							5.4 (0.25)						
**Body image perception**														
Positive body image	23	29.9	39	50.6	15	19.5	77	24	35.3	32	47.1	12	17.6	68
Negative body image	22	30.1	35	47.9	16	21.9	73	26	31.7	37	45.1	19	23.2	82
*χ*^2^ (*p* value)	1.6 (0.9)							0.72 (0.697)						

*χ*^2^ test of significance, *p* < 0.05 (significant)

## Discussion

Despite the fact that selfies are not a new phenomenon, there is still a need for academic research into their relationships with different social and personal data. In recent years, selfies have been related to recording all facets of everyday life (e.g., personal relationship circumstances and private conditions), as well as some spectacular accidents (e.g., Al-Hag in the Islamic religion). As well as a few major mishaps around the world, selfie concepts (self-esteem and body image perception) and behaviors (selfie habits and patterns, the motives for using the selfie, and attitude about selfie) among Egyptian and Saudi nursing students were important to investigate.

In terms of the Saudi research sample’s personal characteristics, results revealed that the majority of Saudi students were in the age ranged 20-22 years. This result might be attributed to the fact that rapid advancements in digital imaging and mobile technology have ushered in a new era of photography for this community of Saudis. The present study finding was consistent with a previous study conducted in Northampton, and found that young women (18 to 29 years old) use Instagram to post selfies in order to gain “likes,” and that the quality of a selfie is determined by lighting [23].

In addition, young individuals (18-34 years old) are more interested in the selfie trend than older people (35 years old and above). The explanation given was that teenagers and people between the ages of 18 and 34 use digital media more than older people [4].

The findings of this study analysis revealed that Egyptian females were more than Saudi females in the study sample. This result indicates that the majority of Egyptian females have a sense of duty, intelligence, and life experience, which leads to a desire and willingness to participate in the research. This study finding match those of the study conducted in KSA, which reported that over the course of 3 months, a total of 653 participants who met the inclusion requirements completed the online questionnaire, with 164 (25.1%) men and 489 (74.9%) women [24]. Consistent with the previous findings, several study results found that female participants are thought to take 1.3 times more selfies than male participants [25].

In responding to the first research question, as regards selfie habits and patterns, Egyptian students were higher in number of selfie taken per day more than Saudi students. This finding might be explained by the fact that people can find the amount of selfies they take and share on a social networking outlet that best fits their desires and allows them to gain gratification.

This finding might be attributed to that the individuals will find the number of selfies taken and posted on a social networking outlet that best fits their desires and allows them to gain gratification. In a similar study, the association between self-esteem and the people who take more selfies at Middle Tennessee State University was investigated, and discovered that persons with lower self-esteem likely to take more selfies than those with higher self-esteem. However, persons with high self-esteem shared the most selfies, according to the findings [26].

Also, the current study result found a statistically significant difference between Saudi and Egyptian nursing students as regards prefer to take their selfies with friends; however, this finding contradicted with previous study found that a total of 2071 British men and women between the ages of 18 and 30 took part in the survey. According to the data, 39% of the individuals preferred to photograph themselves rather than their family, partner, or pets. According to their physical attributes, the individuals had low self-esteem and reported to “prudish inhibition and concerns regarding their physical qualities” [26].

Also, the findings of this study revealed that the majority of Saudis group compared to three-fifths of Egyptians, preferred to post selfies on Instagram, with a statistically significant difference. With 187 million users, this result indicates that Snapchat, an app that allows users to send messages as well as take and share videos and images, is becoming increasingly popular.

In line with this study findings, previous research found that Instagram was a common social network site with about 600 million users and millions of selfie photos posted daily [27]. On the other hand, according to the Pew Center’s 2014-2015 survey, 71% of teens said they use Facebook on a regular basis. Furthermore, there was no other website that a great majority of teenagers used at the time, with around half (52%) using Instagram and 41% using Snapchat [28]. Without a doubt, the data shows that how teenagers use social media is evolving, and it is critical that people are aware of the potential consequences of various channels.

As regards the motives of using selfies, the current study result showed that Egyptian nurse students are having a higher prevalence of subjective desire as a reason for sharing selfies on social media than Saudi nurse students and there was statistically significant difference between the two groups. This could be because the participants shared selfies since getting likes made them feel better. On the contrary, other study found that uploading selfies was amusing in a study conducted at Middle Tennessee State University (15%). It is possible that the participants require some form of media to reduce their stress [1]. Also, a research of a Saudi population found that using social networking sites (SNS) improved users’ social skills and elevated their self-esteem [29].

Furthermore, some people have discovered that posting selfies on social media relieves their stress and thereby entertains them [30]. As well, the use of social media use is a possible cause for appearance pressures and, as a result, appearance-altering practices such as cosmetic surgery [31].

In the present study, reasons for taking selfies were significantly higher in Saudi nursing students than in Egyptian students regarding depression and sadness. The study found that the more time users spent on social media and the more time they spent managing impressions, the more likely they were to develop clinical depressive symptoms. In line with this current study finding, literature suggests a significant relationship between social media use and mental health at Bryant University [32]. According to earlier research in which the association between social media use and depression was investigated, depression is one of the most common side effects of long-term social media use. This link demonstrates the negative effects that long-term social media use can have on users’ mental health [33].

As regards the attitudes of studied nurse students about the selfie, the current study result found that majority of studied Egyptian nurses think that selfie is normal for them if it is frequently depicted in front of people compared with more than half of Saudi students. Studied student nurses from Egyptian and Saudi groups see some people photographing themselves in places for gaining fame with a slightly increasing percent in Egyptian more than Saudi group.

More than half of Saudi students compared with more than two-fifth of Egyptians in their opinions, they see a person who was trying to prove himself through the selfie is someone who likes to impose himself. Majority of studied students in the two groups see that the phenomenon of selfie had become an addiction for some people. According to the opinion of Egyptian nurse students, near half of them see selfie style is so prevalent today because it was fashionable times, however, in Saudi group more than one-third of them see selfie as usual. The difference was statistically significant between the two groups.

These study results agreed with previous study found that students may view selfies as pointless, awkward, and uncool; further, they believe it is an attention-seeking activity [34]. However, this finding contradicts a study published in Egypt, which found that male students have more negative attitudes regarding selfies [35].

In relation to students’ self-esteem, the current study result revealed that more than half of studied Egyptian nursing students were good in the level of self-esteem compared with more than one-third of Saudi group. The difference between two groups was statistically significant. More than half of studied Egyptian nursing students were positive in body image perception; however, in Saudi group, they were negative in perception of their body image. The difference was not statistically significant. These results answered the second research question.

The current study results contradict with those of the study conducted in Egypt who found that 21.4 of studied girls in preparatory schools in Zagazig City were not accepting their body image, while 33.7% of them had low self-esteem [36]. Additionally, other research conducted in Brazil found that more than two-thirds of studied students were not accepting their own body image [37].

As regards relationship between the number of selfie-taking and gender, the current study finding revealed that a statistically significant difference was detected between gender in both Egyptian and Saudi groups with increasing taking selfie among Egyptian females and Saudi males. This result might be due to culture difference between the two groups. Traditional Saudi norms and standards regard women’s visibility as harmful, and tacitly reject their self-presentation through photographs and videos. This finding contradicts the findings of a previous study which found that women snap more selfies than men [38]. As well, a previous research also found that women took 1.3 times more selfies than men [25]. On the contrary, a recent study found that age and gender are irrelevant when it comes to selfies [39].

Considering relationship between sharing selfie on social media and gender, the current result study revealed that a statistically significant difference was detected between genders in both Egyptian and Saudi groups with increasing sharing selfie on social media among males. This might be because men have been observed to be more intrepid and daring in their social presentation and expression across cultures and academic studies. These study findings are consistent with those conducted in Egypt found that males are more likely than females to post selfie photos on social media [35].

Similarly, it was found that in the undergraduate group, more girls were dissatisfied than guys. At the same time, postgraduate males scored much higher in body image acceptance than undergraduate males. People who were more satisfied with their body image posted more confident selfies to Instagram, according to their research [19].

However, these findings contradicted those who investigated selfie posting behaviors in relation to gender and found that female subjects had higher mean scores for posting selfie images than men in their study. Furthermore, in 2014, selfiecity, a project financed by The Graduate Center at City University of New York, evaluated the types of selfie in five cities around the world, concluding that women were more likely than men to share selfie images [38].

The current study result revealed no relationship detected between self-esteem and body image of the studied nurse students in Egyptian and Saudi groups and number of selfie-taking and posting selfie on social media. This answers the third and fourth research questions. One possible explanation for the lack of a significant difference is that persons with high self-esteem posted about the same amount of selfies as persons with low self-esteem. According to previous research, persons with low self-esteem may be just as eager to publish selfies on social media sites as those with greater self-esteem since it provides an alternative to in-person self-disclosure in a secure, controlled context [9]. In the same vein, a study conducted in Ireland and Hong Kong stated that self-esteem had no significant relationship with selfie frequency [40]. Another research also discovered no link between selfitis and self-esteem in Indian medicine and nursing students [41].

In contrast to the current study’s findings, one study conducted in Tennessee showed that persons with low self-esteem took the fewest selfies, while those with high self-esteem took the most [1]. In a similar study, the researcher explained the reduction in selfies by claiming that individuals did not appreciate seeing images of themselves with less-than-attractive bodies on social media platforms such as Facebook, Twitter, and Instagram [42]. Another reason for the drop in selfies could be their poor self-confidence, which was discovered in a study titled “Selfies and their Psychological and Mental Effects” [43]. Several prior studies in this field have found that using selfies enhances one’s self-esteem, self-confidence, and popularity [44, 45]. As well, a more recent research found that the selfie habit of the students can boost their self-esteem [46].

## Conclusions

The current study deduced that Egyptian students as regard selfie habits and patterns had a higher number of selfies per day than Saudi students, also the majority of the Saudi group as regard selfie habits like to put their selfies on Instagram with a statistically significant difference. As regards the motives for using the selfie, the reasons for taking selfies were significantly higher in Saudi nursing students than in Egyptian students regarding depression and sadness. As well, significantly higher in Egyptian than in Saudi nurse students was related to motivate that their selfies on social media often contain comments or answers, the relationship between the number of selfie-taking and gender was a statistically significant difference between the two genders in both Egyptian and Saudi groups with increasing taking selfie among Egyptian females and Saudi males.

The current study’s findings do not suggest that selfie is a mental disorder; rather, they suggest that selfie looks to be a condition that requires further investigation to fully assess the psychosocial consequences of the selfie phenomenon on behavior. Students can recognize that snapping selfies has an impact on their self-esteem based on the findings of the study. As a result, researchers anticipate that this finding will bring new insight into how people interact with, interpret, and are impacted by their selfies in social computing and technology-mediated environments.

## Data Availability

As detailed in the “References” section, all data was accessible through the Internet.

## Abbreviations

KSA: Kingdom of Saudi Arabia
SCQ: Self-confidence questionnaire
